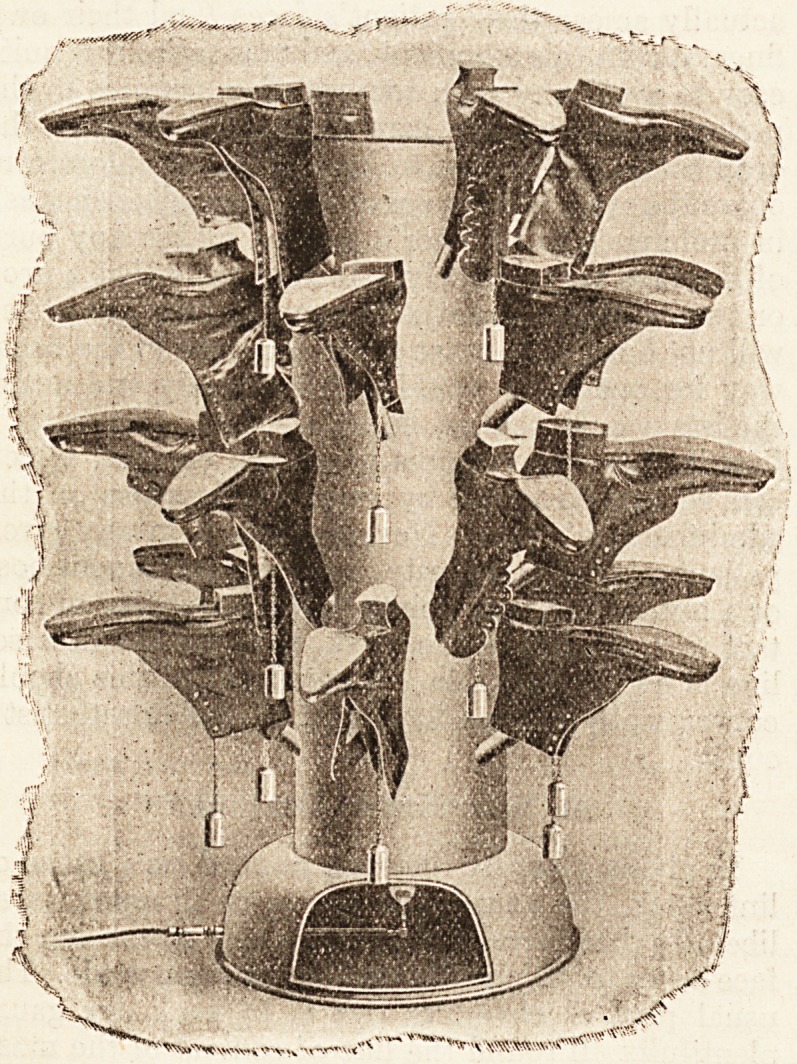# New Appliances and Things Medical

**Published:** 1910-01-01

**Authors:** 


### NEW APPLIANCES AND THINGS MEDICAL.

[We shall be glad to receive at our Office, 28 & 29 Southampton Street, Strand, London, W.C., from the manufacturers, specimens of all new preparations and appliances.]

### THE HOT AIR BOOT DRYER.

The accompanying illustration gives a better idea of what the hot-air boot-drier, manufactured by Fisher and Ludlow, Ltd., of Albion Works, Birmingham, is like than any verbal description can do. It is a thoroughly sensible invention, and is the only way we know of drying wet boots without injuring the leather. As of most good things, the principle of the hot-air boot-drier is simple, and consists in drying the boots from the inside with hot air in the manner illustrated. Boots that are dried on the feet in front of the fire scorch and twist the leather, besides driving the damp into the inside, with the result, as we all know, of another pair of boots ruined. None of these evils attend this invention, which, although it is a process of several hours, will not harm the boots if continued well nigh indefinitely.

### IMPROVED SANITARY APPLIANCES.

Messrs. Doulton and Co. have sent us particulars of some improved sanitary appliances they are introducing. The apparatus are all made of the firm's well-known pottery, with the usual accessory woodwork. In one of the appliances a white "Queensware" valve closet of the usual form is placed above a pedestal of the same material, in which the trap is enclosed, so as to be above the floor line. The closet has a double thickness polished mahogany seat, and the regulating supply valve and outlet valve are worked by one lever at the side. The rubber seating to the outlet valve can be replaced without removing the pan. In another apparatus on the same lines, the pan is flushed and the contents discharged by pressing a push fixed on the wall just above. In an appliance particularly suited for hospitals and kindred institutions the pan is enclosed inside a removable box of Queensware, the pan being discharged and flushed by means of a handle at the side. There is the usual mahogany seat, with mahogany back. The enclosing ware box can be removed at any time easily and quickly for cleaning purposes. The rubber seating of the outlet valve of this apparatus can be replaced also without removing the basin. A fourth appliance is arranged on the armchair plan. The pan is of the usual form, enclosed inside a Queensware box, instead of wood. The armchair is movable, so that the floor can be got at for cleaning. The appliance has a slop top in one piece with the pan. The back of the chair is of double cane. There is a pull-up knob at the side for discharging and flushing. This appliance has been fitted in a number of mansions. The whole of the apparatus are finished in Messrs. Doulton's well-known style, and are supplied from their Lambeth works.

## Figures and Tables

**Figure f1:**